# Supplementary material for: Passive broadband full Stokes polarimeter using a Fresnel cone
Source: Sci Rep. 2019 Feb 25;9:2688. doi: 10.1038/s41598-019-39118-0 (PMC6389910; doi:10.1038/s41598-019-39118-0)
Supplement: Supplementary file 1 — Supplementary Material [file 41598_2019_39118_MOESM1_ESM.pdf]

Passive broadband full Stokes polarimeter using a Fresnel cone: supplementary material

R. D. Hawley*[1], J. Cork[1], N. Radwell[1], and S. Franke-Arnold[1]

[1]SUPA and School of Physics and Astronomy, University of Glasgow, Kelvin Building, Renfrewshire, Glasgow, G12 8QQ, UK
*Corresponding author: r.hawley.1@research.gla.ac.uk

ABSTRACT

Here we present the Mueller matrices for the Fresnel cone polarimeter system. We show the method for normalisation of the measured Mueller matrices for the beam-splitter in the system and give an example of the resulting linear system.

1 Mueller matrices

The Mueller matrices used in Equation (1) are

$$\mathbf{R}(\theta) = \begin{bmatrix} 1 & 0 & 0 & 0 \\ 0 & \cos(2\theta) & \sin(2\theta) & 0 \\ 0 & -\sin(2\theta) & \cos(2\theta) & 0 \\ 0 & 0 & 0 & 1 \end{bmatrix}, \tag{1}$$

$$\mathbf{R}(-\theta) = \begin{bmatrix} 1 & 0 & 0 & 0 \\ 0 & \cos(2\theta) & -\sin(2\theta) & 0 \\ 0 & \sin(2\theta) & \cos(2\theta) & 0 \\ 0 & 0 & 0 & 1 \end{bmatrix}, \tag{2}$$

$$\mathbf{M}_{\mathrm{qwp}} = \begin{bmatrix} 1 & 0 & 0 & 0 \\ 0 & 1 & 0 & 0 \\ 0 & 0 & 0 & 1 \\ 0 & 0 & -1 & 0 \end{bmatrix}, \tag{3}$$

and

$$\mathbf{M}_{\mathrm{pol}} = \frac{1}{2}\begin{bmatrix} 1 & 1 & 0 & 0 \\ 1 & 1 & 0 & 0 \\ 0 & 0 & 0 & 0 \\ 0 & 0 & 0 & 0 \end{bmatrix}. \tag{4}$$

2 Mueller matrix measurement and normalisation

Polarisation state recovery is achieved by solving Equation (9) for $\mathbf{S}$, where Equation (9) is

$$\underbrace{\begin{bmatrix} 1 & \frac{1}{2} & 0 & 0 \\ 0 & 0 & 0 & -\frac{1}{2} \\ 0 & \frac{1}{2} & 0 & 0 \\ 0 & 0 & \frac{1}{2} & 0 \end{bmatrix}}_{\mathbf{M}} \underbrace{\begin{bmatrix} s_0 \\ s_1 \\ s_2 \\ s_3 \end{bmatrix}}_{\mathbf{S}} = \underbrace{\begin{bmatrix} a_0 \\ b_2 \\ a_4 \\ b_4 \end{bmatrix}}_{\mathbf{C}}. \tag{5}$$

While this is true for idealised systems, real optical components such as beam-splitters introduce unwanted polarisation shifts. We can measure and account for these unwanted polarisation shifts by characterising the Mueller matrices of our physical

system. These Mueller matrices are obtained from 36 intensity measurements while generation and analysing the six basis polarisation states (H,V,A,D,R and L) using the method outlined in[1].

Without careful normalisation of these measured Mueller matrices, the polarisation state recovery can return non-physical Stokes vectors. We attribute this to systematic errors in the Mueller matrix measurements. To avoid these non-physical Stokes vectors we choose to normlaise our Mueller matrices in the form of Equation (5) in Ref.[2];

$$\mathbf{M} = \begin{bmatrix} 1 & a & b & 0 \\ a & u & v & -x \\ b & v & w & y \\ 0 & x & -y & p \end{bmatrix}, \tag{6}$$

where

$$\begin{aligned} p &= \sin(2\psi)\cos(\delta) \cong 0, \\ r &= \sin(2\psi)\sin(\delta) \cong 1, \\ s &= \cos(2\psi) \cong 0, \\ a &= s\cos(2C), \\ u &= f\cos(4C) + (1-f), \\ b &= s\sin(2C), \\ v &= f\sin(4C), \\ x &= r\sin(2C), \\ w &= -f\cos(4C) + (1-f), \\ y &= r\cos(2C), \\ f &= (1-p)/2 \backsimeq 0.5. \end{aligned} \tag{7}$$

This normalisation assumes no loss of polarisation in the beam-splitter and that the Mueller matrix is parameterised by three angles, namely a phase-shift induced between s and p polarisation components (δ), a rotation of the linear polarisation components (ψ), and an orientation of the beam-splitter from horizontal (C). We calculate the above matrix $\mathbf{M}$ for 300 values of the three angles between 0 and π. We find the sum of the squared difference between each calculated Mueller matrix and our measured matrix, and choose the matrix with the smallest difference. We do this to find $\mathbf{B}_{\text{refl}}$ and $\mathbf{B}_{\text{trans}}$ for each of the red, green and blue colour channels independently.

Using these normalised Mueller matrices in the analysis results in $\mathbf{M}$ becoming a 5×4 matrix and $\mathbf{C}$ gaining an additional term. For example, for the red frequency band Equation (S7) becomes

$$\underbrace{\begin{bmatrix} \frac{31}{237} & \frac{52}{1301} & -\frac{1}{23754} & \frac{1}{1176} \\ \frac{1}{10822} & -\frac{1}{38210} & -\frac{1}{300} & -\frac{1}{430} \\ \frac{1}{460} & \frac{1}{1453} & -\frac{5}{203} & -\frac{9}{118} \\ \frac{1}{1944} & \frac{22}{551} & \frac{1}{322} & \frac{1}{6482} \\ -\frac{1}{1452} & -\frac{1}{480} & \frac{13}{310} & -\frac{3}{322} \end{bmatrix}}_{\mathbf{M}} \underbrace{\begin{bmatrix} s_0 \\ s_1 \\ s_2 \\ s_3 \end{bmatrix}}_{\mathbf{S}} = \underbrace{\begin{bmatrix} a_0 \\ a_2 \\ b_2 \\ a_4 \\ b_4 \end{bmatrix}}_{\mathbf{C}}, \tag{8}$$

where

$$a_2 = \frac{4}{N}\sum_{i=1}^{N} I_i \cos 2\theta_i \quad . \tag{9}$$

The pseudo-inverse of $\mathbf{M}$ is then calculated using the in-built LabVIEW function which is based on the singular value decomposition method.

References

1. H. Fujiwara, *Spectroscopic ellipsometry: Principles and applications* (Wiley-Blackwell, 2007).

2. P. S. Hauge, "Mueller matrix ellipsometry with imperfect compensators," J. Opt. Soc. Am. **68**, 1519 (1978).
